# Pathological mechanism of heart failure with preserved ejection fraction in rats based on iTRAQ technology

**DOI:** 10.7717/peerj.15280

**Published:** 2023-05-03

**Authors:** Hang Xu, Kai Gao, Chao Liu, Tian Li, Yi Ding, Jing Ma

**Affiliations:** 1 Department of Traditional Chinese Medicine, Xijing Hospital, Fourth Military Medical University, Xi’an, Shaanxi, China; 2 Department of Pharmacy, Xijing Hospital, Fourth Military Medical University, Xi’an, Shaanxi, China; 3 Department of Cardiology, Xijing Hospital, Fourth Military Medical University, Xi’an, Shaanxi, China; 4 School of Basic Medicine, Fourth Military Medical University, Xi’an, Shaanxi, China

**Keywords:** Heart failure with preserved ejection fraction, Pathogenesis, Differentially expressed protein, PPAR, iTRAQ technology

## Abstract

**Objective:**

Heart failure with preserved ejection fraction (HFpEF) is a public health problem worldwide. Treatments for the patients with HFpEF are not satisfactory because there is no unified understanding of the pathological mechanism of HFpEF. This study aims at investigating the potential pathological mechanism for the effective diagnosis and treatment of HFpEF.

**Methods:**

Ten adult male Dahl salt sensitive rats (180–200 g) were divided into control and model groups. The rats in model group were fed with high salt diet (8% NaCl) to induce HFpEF for this comparative study. Behavioral changes, biochemical parameters, and histopathological changes of the rats were detected. iTRAQ technology combined with bioinformatics analysis was employed to study the differentially expressed proteins (DEPs) and their enrichment in signaling pathways.

**Results:**

Echocardiography detection showed decreased LVEF, indicating impaired cardiac function (*P* < 0.01), increased LVPWd, indicating ventricular wall hypertrophy (*P* < 0.05), prolonged duration of IVRT and decreased E/A ratio, indicating diastolic dysfunction (*P* < 0.05) of the rats in model group. 563 DEPs were identified in the rats of both groups, with 243 up-regulated and 320 down-regulated. The expression of PPAR signaling pathway in the rats of model group was down-regulated, with PPAR*α* most significantly decreased (91.2%) (*P* < 0.01), PPAR*γ* obviously decreased (63.60%) (*P* < 0.05), and PPAR*β*/*δ* decreased (45.33%) (*P* < 0.05). The DEPs enriched in PPAR signaling pathway were mainly related to such biological processes as fatty acid beta-oxidation, such cellular components as peroxisome, and such molecular functions as lipid binding.

**Conclusions:**

NaCl high salt diet is one of the factors to increase the incidence of HFpEF in rats. PPAR*α*, PPAR*γ* and PPAR *β*/*δ* might be the targets of HFpEF. The findings may provide a theoretical basis for the treatment of HFpEF in clinical practice.

## Introduction

Heart failure (HF) is classified into five types according to left ventricular ejection fraction (LVEF) assessment in universal definition and classification of heart failure in 2021 (Bozkurt et al., 2021). They are HF with reduced EF (HFrEF), HF with mildly reduced EF (HFmrEF), HF with preserved EF (HFpEF), HF with improved EF (HFimpEF), and HF with supernormal ejection fraction (HFsnEF). Among them, HFpEF accounts for more than 40% of HF patients with high mortality and morbidity (Lewis et al., 2018; Severino et al., 2021; Withaar et al., 2021). The majority of HFpEF patients are elderly people accompanied by such complications as hypertension, atrial fibrillation and renal insufficiency. They suffer from impaired left ventricular diastolic function, increased stiffness and pulmonary congestion, leading to dyspnea, pulmonary hypertension and exercise intolerance (Becher et al., 2013; Borlaug, 2014; Dunlay, Roger & Redfield, 2017). Since HFpEF has different clinical phenotypes and complex pathophysiological mechanism (Ozlek et al., 2019), HFpEF patients are currently considered as the largest “unmet needs” in cardiology (Pfeffer, Shah & Borlaug, 2019). Therefore, in-depth study of HFpEF pathological mechanism is essential for the effective treatment of HFpEF.

Previous studies have explored the pathogenesis of HFpEF based on epidemiology, pathophysiology, and cytology. Studies have found that hypertension is a main cause of HFpEF (McKee et al., 1971) and HFpEF is mainly correlated with left ventricular diastolic dysfunction, left ventricular systolic dysfunction, left atrial dysfunction, pulmonary hypertension, right ventricular dysfunction, coronary microvascular dysfunction, vascular and microvascular dysfunction, and abnormal peripheral and cardiac amyloidosis (Pfeffer, Shah & Borlaug, 2019). In transgenic mice which express catalase in mitochondria, the HFHS diet-induced mitochondrial abnormalities, left ventricular hypertrophy and diastolic dysfunction were ameliorated, indicating that mitochondrial reactive oxygen species may cause mitochondrial dysfunction to a certain extent (Sverdlov et al., 2016). In high fat and high sugar MHD mice, reduced ATP production results in functionally important energy deficiency and increased ADP while maintaining CK flux (Luptak et al., 2018). Nowadays there is no unified understanding of the pathogenesis of HFpEF and there are limitations in the understanding of HFpEF by clinicians (Brouwers et al., 2013). With the development of proteomics, it is necessary to take full play of the new science and investigate the pathological mechanism of HFpEF from a new perspective.

The isobaric tags for relative and absolute quantification (iTRAQ)-labeling are one of the most reliable methods to make quantitative analysis of proteins based on peptide identification (Wang et al., 2022b). iTRAQ technology has been widely used in cardiovascular medicine in recent years but there are few reports of its application in HFpEF. We established a HFpEF rat model using high salt diet to make a comparison with healthy rats. iTRAQ technology combined with KEGG enrichment and GO annotation analysis was employed to study the pathogenesis of HFpEF. Our study aimed at providing a potential pathological mechanism for the effective diagnosis and treatment of HFpEF.

## Materials and Methods

### Animals and grouping

Experiments were performed on 10 adult male Dahl salt sensitive rats aged 8 to 12 weeks and weighing 180 to 200 g. They were purchased from Beijing Vital River Experimental Animal Science and Technology Co., Ltd. (SCXK (Beijing) 2016-0006). The rats were placed in an animal chamber at room temperature 25 °C ±2 °C, air humidity 50%–70%, and light/dark cycle for 12 h, and were given free access to food and water. After 7 days of adaptive feeding, the rats were randomly divided into control group and model group (*n* = 5). The rats in two groups were fed with 0.3% NaCl diet and 8% NaCl high salt diet for 8 weeks, respectively (Gallet et al., 2016). A HFpEF rat model was established and met these criteria (LVEF ≥50%, BNP>100 pg/ml, and NT-ProBNP>300 pg/ml), indicating the presence of HFpEF in model group. The animal experiment was performed with the formal approval of the Medical Ethics Committee of the First Affiliated Hospital of the Fourth Military Medical University (FE15611-07) and was in line with the Guidelines for the Management and Use of Laboratory Animals by the Chinese National Institutes of Health.

### Measurement of body weight, systolic and diastolic blood pressure, heart weight, heart weight/body weight, heart weight/tibia length

The body weight of the rats was measured by electronic scale once a week for eight weeks to observe the body weight changes of the rats. Non-invasive blood pressure testing was conducted to measure the values of systolic and diastolic blood pressure of the rats in both groups. The test was performed once a week for eight weeks. The rats were placed on a preheated thermostatic testing table and induced into a black fixation box to expose the caudal sacral region. The pressure transducer of the non-invasive sphygmomanometer was snapped into the artery at the tail root and the tail sleeve was connected to a compressed air cylinder *via* an inlet and exhaust valve arrangement to allow continuous inflation and exhaust of the cuff. The cuff pressure was continuously recorded with a fixed pressure transducer. The recording was started by connecting the detection device. The systolic and diastolic blood pressure values of the rats in both groups were continuously recorded 20 times with the average of the last 10 data for statistical analysis. On day 59 of the experiment, the rats in two groups were sacrificed to measure their heart weight, heart weight/body weight, heart weight/tibia length.

### Measurement of running and swimming exhaustion time

The running and swimming exhaustion experiments were performed to observe the physical changes of the rats after eight weeks of feeding. The running and swimming exhaustion exams were conducted at 24 h intervals to ensure the recovery of the rats’ physiological functions, *i.e.,* on day 57 and 58, respectively. There was 24 h interval for exercise exams and sacrifice time of the rats. The rats in both groups were placed on the treadmill and ran at 20 m/min until they reached the exhaustion standard (*i.e.,* righting reflex disappeared). On the 2nd day of the running experiment, the rats were placed in a water tank (water temperature 28 ± 3 °C, water depth 50 cm, length and width 50 × 50 ×80 cm) and swam until they reached the exhaustion standard (*i.e.,* their heads were submerged in water for 5 s and could not rise to the surface).

### Detection of cardiac functions

After 8 weeks of feeding, the differences in cardiac functions of the rats in two groups were detected by echocardiography ($\bar {\mathrm{x}}\pm $s, *n* = 5). The rats were anesthetized through isoflurane inhalation. They were fixed in supine position on a table with their hair on the left chest shaved. HR, LVEF, LVID, LVPW, E/A, IVRT, and IVCT of the rats were detected by an ultrasound imaging system (RMV707B probe with a frequency of 30MHz and a detection depth of 12–16 mm) with a 15L8 ultrasound probe. The probe was placed on the left chest of the rats and the left ventricular motion was recorded by M-mode ultrasound. Three different cardiac cycles were selected for each parameter to get the average. We were blind to the rats in two groups when performing echocardiography and making echocardiography analysis. Vevo LAB 3.1.1 software was used for data analysis.

### ELISA detection

After the blood of the rats was taken from abdominal aorta by vacuum vessels, it was put on ice and centrifuged within 30 m. Serum was collected and stored at −80 ° C for use. Then brain natriuretic peptide (BNP) was detected by ELISA kit (sensitivity: 0.75 pg/ml; Sigma-Aldrich, St. Louis, MO, USA) and N terminal pro B type natriuretic peptide (NT-proBNP) was detected by ELISA kit (sensitivity: 0.81 pg/ml; Abcam, Waltham, MA, USA). Each sample was repeated three times with the average value as the result. OD value of absorbance was set as the ordinate (Y) and the corresponding standard concentration was set as the abscissa (X) to draw the standard curve. The sample content was calculated in line with OD value (expressed as pg/ml) and the sample concentration was analyzed based on the standard curve.

### HE staining and Masson staining

Heart tissues of the rats were administered with HE staining and Masson staining according to previous work (Wang et al., 2022a). In short, heart tissues were cut with pathological methods and were observed with a microscope. At least five fields of view of each rat were observed for qualitative analysis of HE staining. Eight independent Masson stained section images (×200, bar = 50 µm) were selected to calculate collagen volume fraction (CVF) by Image-Pro Plus for the assessment of cardiac fibrosis.

### iTRAQ analysis

**Figure 1:**
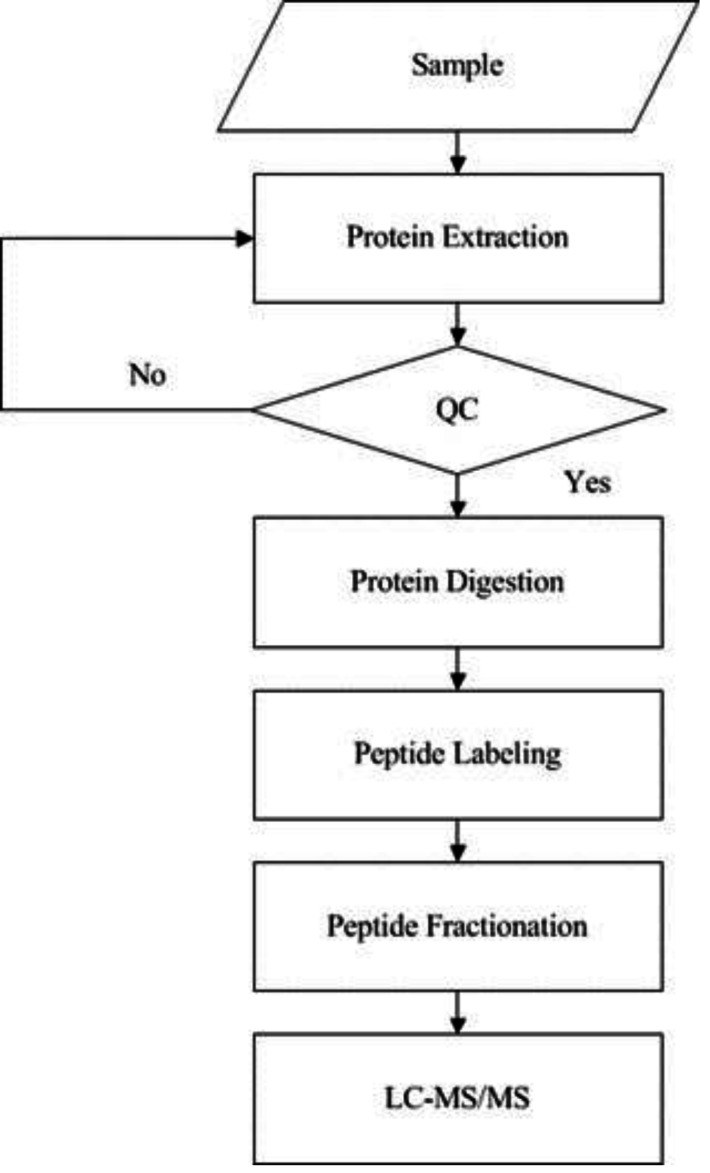
Experimental procedures of iTRAQ quantitative proteomics. The supernatant of the sample was obtained by protein extraction and the protein concentration of the sample was calculated according to the standard curve and sample OD595 after quantification by Bradford. Then the protein concentration of the sample was determined by SDS-PAGE, protease hydrolysis, peptide labeling, peptide separation, high performance liquid phase, and mass spectrometry sequentially.

iTRAQ technology was applied to screen and identify DEPs of the rats in control group and model group. Analyses were made according to the established procedures in our laboratory (Xu et al., 2019) (Fig. 1). The main procedures of iTRAQ technology consists of five steps: protein extraction, protein digestion, peptide labeling, peptide fractionation, and LC-MS/MS analysis. Briefly, myocardial tissues were proteolytically cleaved, myocardial proteins extracted by reductive alkylation were enzymatically enriched, and protein concentrations were determined by the Brandford method and detected by electrophoresis on polyacrylamide gels. After equal amounts of Tripsin were digested from each sample, peptides were labeled with iTRAQ reagent and mixed in equal amounts and pre-separated by SCX. The raw mass spectral data were analyzed by LC-MS/MS.

The raw mass spectrometry data were converted to MGF format by ProteoWizard tool msconvert, and then the converted MGF files were compared and searched by Mascot 2.3.02 and the selected protein sequence database to get the final protein identification results. IQuant software was used to filter the proteins, correct the gene tags for purity, normalize the quantified values, complete the missing values, calculate the quantified protein values, and analyze the statistical tests to display the quantitative protein iTRAQ data. A total of 15,834 peptides and 3,690 proteins were identified in the iTRAQ quantification of myocardial tissue proteins under 1% FDR.

### KEGG enrichment analysis and GO annotation of DEPs

DEPs in the rats of control and model groups were identified according to Log2—Fold Change—>1.2 standard. KEGG enrichment analysis and GO annotation of DEPs were conducted using BGI analysis system (https://biosys.bgi.com/#/report/login). KEGG enrichment analysis was made for the relation of DEPs with signaling pathways. GO annotation analysis was made to classify the DEPs in PPAR signaling pathway into biological process, cellular component and molecular function.

### Western blotting

The expressions of PPAR *α*, PPAR *β*/ *δ* and PPAR *γ* in heart tissues of the rats were analyzed by Western blotting according to previous method (Li et al., 2020). After sealed, the membrane was combined with PPAR *α* Polyclonal antibody at dilution of 1:600 (15540-1-AP, Wuhan Sanying Biotechnology Co., Ltd), Anti-PPAR *β*/ *δ* Rabbit pAb at dilution of 1:1000 (GB113450, Wuhan Sewell Biotechnology Co., Ltd.) and PPAR *γ* Polyclonal antibody at dilution of 1:5000 (16643-1-AP; Wuhan Sanying Biotechnology Co., Ltd.) as primary antibody, respectively and it was incubated at 4 ° C overnight. Anti-GAPDH was used for loading control (10494-1-AP, Wuhan Sanying Biotechnology Co., Ltd). Then the membrane coupled with the secondary antibody (HRP, A21020; Wuhan Abbkine Biotechnology Co., Ltd.) was incubated at room temperature for 1.5 h. Enhanced chemiluminescence (ECL) was used to detect protein band. ImageJ was applied to read the band grey value.

### Statistical analysis

The experimental data were analyzed by SPSS 22.0 (Chicago, USA) and were presented as the mean ± standard deviation. Comparison between two groups was made by two-tailed Student’s *t*-test. Statistical significance was considered at *P* < 0.01 or *P* < 0.05.

## Results

### Changes in body weight, systolic and diastolic blood pressure, heart weight, heart weight/body weight, heart weight/tibia length of the rats

**Figure 2:**
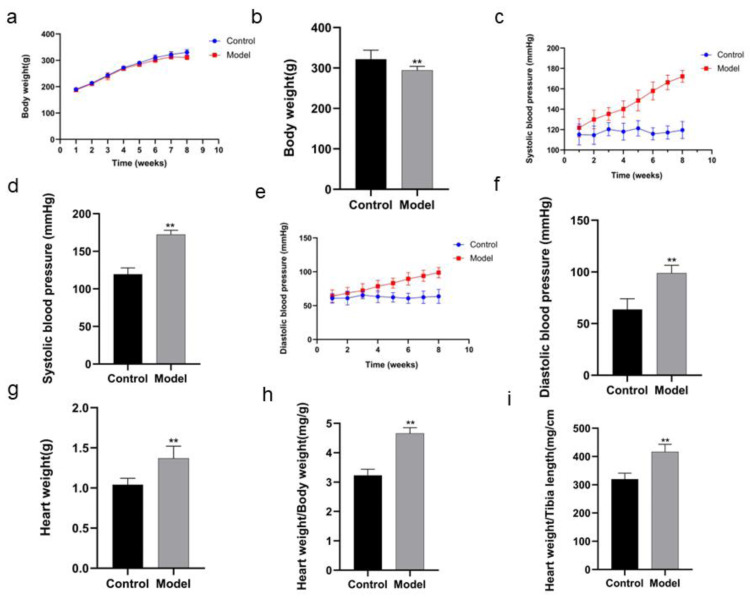
Comparison of indexes in the rats of two groups. In body weight (A), running exhaustion time (B), swimming exhaustion time (C), echocardiography detection (D), HE staining (E), and Masson staining (F). Scale bar = 40 µm.

The body weight of the rats in model group was decreased in the 8th week compared with that in control group (*P* < 0.01) (Figs 2A, 2B; Table 1). After the rats in model group received high salt feeding for 8 weeks, their systolic and diastolic blood pressure values showed a gradual upward trend compared with those of the rats in control group. There were significant differences in systolic and diastolic blood pressure values between two groups on day 56 of the experiment (systolic blood pressure *P* < 0.01, diastolic blood pressure *P* < 0.01) (Figs. 2C, 2D, Table 2; Figs. 2E, 2F, Table 3). On day 59 of the experiment, heart weight, heart weight/body weight and heart weight/tibial length of the rats in control group were significantly increased compared with those of the rats in control group (*P* < 0.01) (Figs. 2G, 2H, 2I).

**Table 1:** Body weight changes of the rats in two groups ($\bar {x}\pm $ s, *n* = 5).

Week	Control	Model
1	188.4 ± 7.82	196.8 ± 20.68
2	214.6 ± 9.31	219.0 ± 19.89
3	242.2 ± 3.70	249.4 ± 18.92
4	257.4 ± 11.37	261.6 ± 16.47
5	283.2 ± 19.21	285.2 ± 10.53
6	297.6 ± 19.91	296.8 ± 8.46
7	306.0 ± 21.43	300.0 ± 5.43
8	321.8 ± 22.42	294.0 ± 10.04^**^

**Notes.**

** *P* < 0.01 *vs.* control.

**Table 2:** Systolic blood pressure of the rats in two groups (mmHg) ($\bar {x}\pm s,n=5$).

Week	Control	Model
1	115.22 ± 10.15	121.86 ± 8.83
2	114.58 ± 9.08	129.97 ± 9.18^**^
3	120.34 ± 6.47	135.36 ± 6.25^**^
4	118.05 ± 8.25	140.13 ± 7.98^**^
5	121.32 ± 7.54	148.55 ± 10.18^**^
6	115.89 ± 5.96	157.97 ± 8.81^**^
7	117.21 ± 6.54	166.26 ± 7.17^**^
8	119.53 ± 8.31	172.23 ± 5.79^**^

**Notes.**

** *P* < 0.01 *vs.* control.

**Table 3:** Diastolic blood pressure of the rats in two groups (mmHg) ($\bar {x}\pm $s, *n* = 5).

Week	Control	Model
1	60.82 ± 7.07	63.91 ± 9.32
2	61.09 ± 10.15	68.58 ± 8.25
3	65.54 ± 5.21	72.25 ± 10.23
4	63.32 ± 8.78	78.54 ± 8.78^**^
5	62.28 ± 6.86	83.19 ± 7.36^**^
6	60.75 ± 7.38	89.52 ± 9.32^**^
7	62.47 ± 9.11	93.91 ± 8.21^**^
8	63.70 ± 10.30	98.82 ± 7.63^**^

**Notes.**

** *P* < 0.01 *vs.* control.

### Changes in running and swimming exhaustion time of the rats

**Figure 3:**
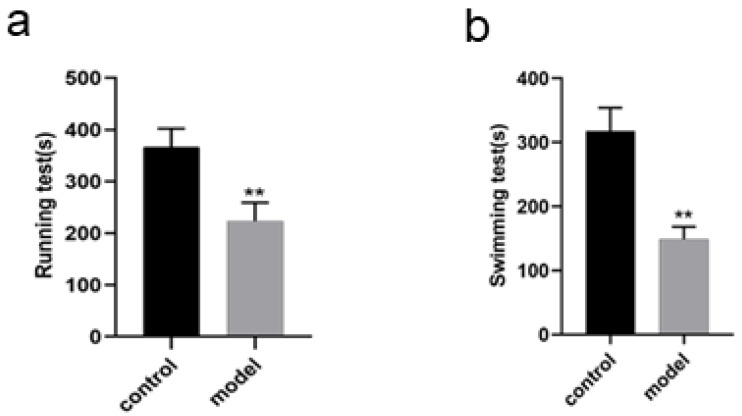
Identification of DEPs by iTRAQ technology, KEGG enrichment analysis and GO annotation of DEPs in PPAR signaling pathway in rats. Up-regulation and down-regulation of identified DEPs (A), Signaling pathways of DEPs (B), biological process of DEPs (C), cellular components of DEPs (D), molecular function of DEPs (E).

The running exhaustion time of the rats in model group was shortened compared with that in control (*P* < 0.01, Fig. 3A) while the swimming exhaustion time of the rats in model group was greatly shortened compared with that in control (*P* < 0.01, Fig. 3B), indicating the changes of the rats in exercise tolerance.

### Changes in cardiac functions of the rats

**Figure 4:**
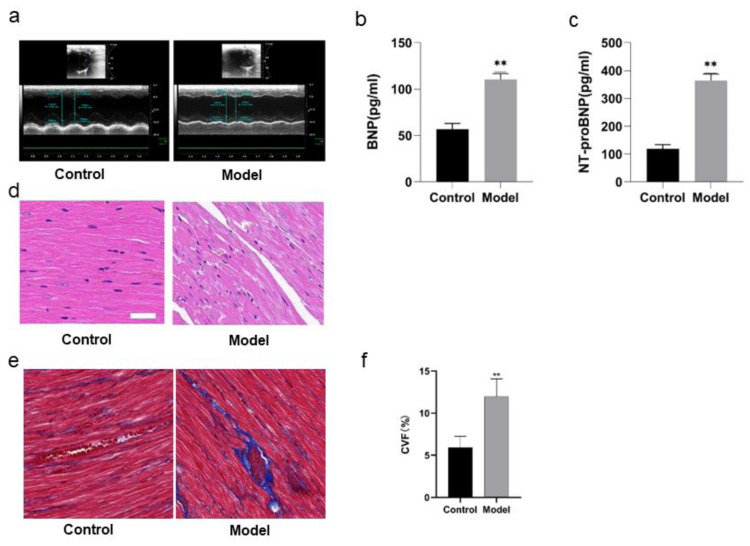
Validation of PPAR signaling pathway in rats by Western blotting. Protein expressions of PPARs in rats of each group (A), PPAR *α* (B), PPAR *γ* (C), PPAR *β*/ *δ* (D). ^∗∗^*P* < 0.01, ^∗^*P* < 0.05 *vs* control group (*n* = 5).

The cardiac functions of the rats were evaluated before the rats were divided into model group and control group. With no difference of HR as condition, the results of echocardiography showed no significant differences in LVEF, LVID, LVPW, E/A, IVRT, IVCT, indicating that there were no differences in the cardiac functions of the rats before intervention. In comparison with the rats in control group, the rats in model group showed some differences after 8 weeks of high salt feeding. The results of echocardiography showed decreased LVEF, indicating impaired cardiac function (*P* < 0.05), increased LVPWd, indicating ventricular wall hypertrophy (*P* < 0.05), prolonged duration of IVRT and decreased E/A ratio, indicating diastolic dysfunction (*P* < 0.05) (Fig. 4A; Table 4). However, there were no significant differences of the rats between two groups in IVCT, indicating no obvious injury of systolic function, and LVID, indicating no enlargement of the heart cavity. These results suggest that a successful HFpEF rat model has been established with diastolic dysfunction as main symptoms.

### Changes in biochemical indexes of the rats

ELISA detection showed that there was a remarkable difference in BNP and NT-proBNP in the rats of two groups. BNP and NT-proBNP of the rats in model group were significantly increased compared with those in control group (*P* < 0.01) (Figs. 4B, 4C; Table 5).

### Pathological changes in heart tissues of the rats

Figure 4D shows HE staining results of heart tissues of the rats in two groups. The myocardial fibers of the rats in control group were arranged in parallel, with regular and dense nuclei of the same size and regular distribution of blood vessels between myocardial fibers, mostly in thin-walled lacunae, lined with flattened endothelial cells. By contrast, the myocardium of the rats in model group was atrophied, with slightly disordered arrangement of myocardial fibers, enlarged myocardial gaps, different sizes of myocardial nuclei, proliferation of blood vessels in the myocardial gaps, dilatation of some lumens and irregular morphology.

**Table 4:** Cardiac functions of the rats in two groups by echocardiography ($\bar {x}\pm $s, *n* = 5).

Group	*n*	HR (BPM)	LVEF (%)	LVID (mm)	LVPW (mm)	E/A	IVRT (ms)	IVCT (ms)
Control(0 week)	5	325.53 ± 12.29	78.63 ± 6.78	7.25 ± 0.38	2.76 ± 0.19	1.26 ± 0.28	18.2 ± 3.89	17.98 ± 5.98
Model(0 week)	5	323.12 ± 9.21	75.32 ± 5.73	7.23 ± 0.50	2.75 ± 0.22	1.23 ± 0.31	18.52 ± 5.15	18.06 ± 4.57
Control(8 weeks)	5	329.4 ± 17.32	74.97 ± 3.41	7.16 ± 0.49	2.77 ± 0.53	1.25 ± 0.32	18.37 ± 4.56	18.02 ± 6.12
Model(8 weeks)	5	326.6 ± 8.16	53.71 ± 2.16^**^	7.22 ± 0.28	2.98 ± 0.31^*^	1.03 ± 0.23^*^	20.82 ± 3.11^*^	18.12 ± 5.63

**Notes.**

* *P* < 0.05.

** *P* < 0.01 *vs.* control.

**Table 5:** BNP and Nt-proBNP of the rats in two groups by ELISA ($\bar {x}\pm $s, *n* = 5).

Group	*n*	BNP	NT-ProBNP
Control	5	57.3855 ± 4.0792	115.5455 ± 9.9917
Model	5	110.7114 ± 5.1450^*^	368.8182 ± 32.0747^*^

**Notes.**

* *P* < 0.05 *vs.* control.

Figures 4E and 4F show the Masson staining observation of heart tissues of the rats in two groups. Myocardial cells of the rats in control group were neatly arranged and a few blue collagen fibers were observed in the interstitium of cells and around blood vessels. However, a lot of collagen fibers were distributed in the myocardial interstitium and many interwoven collagen fibers were observed around blood vessels of the rats in model group. The collagen fibers were increasingly accumulated and the cardiac fibrosis was significantly aggravated (*P* < 0.01).

### Identification of DEPs in rats

**Figure 5:**
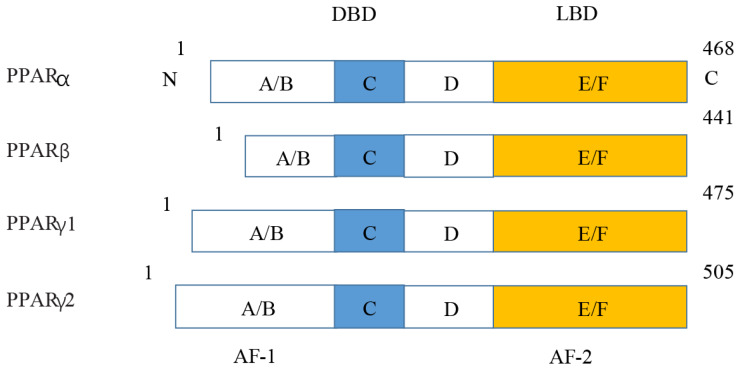
Various structures of the subtypes of PPARs. AF-1/2, activation function-1/2; DBD, DNA-binding domain; LBD, ligand-binding domain.

A total of 563 DEPs of the rats were identified from control and model groups (Fig. 5A). Among them 243 proteins including Podn, LOC498155, Sh3kbp1, Anxa7, Gfpt2 and Bsg were significantly up-regulated while 320 proteins such as Eng, Clu, App, Bpgm5, LOC103689996 and Mrps18b were down-regulated.

### KEGG enrichment and GO annotation of DEPs in PPAR signaling pathway in rat

KEGG enrichment analysis showed that DEPs were most closely associated with PPAR signaling pathway, including its three subtypes PPAR *α*, PPAR *β*/ *δ* and PPAR *γ*. In addition, they were related to such signaling pathways as complement and coagulation cascades, biosynthesis of unsaturated fatty acids, primary bile acid biosynthesis, fatty acid degradation, ascorbate and aldarate metabolism, arachidonic acid metabolism, retinol metabolism, insect hormone biosynthesis, and fatty acid metabolism (Fig. 5B). GO annotation analysis showed that these DEPs enriched in PPAR signaling pathway were mainly related to biological processes such as fatty acid (FA) beta-oxidation and fatty acid metabolic process (Fig. 5C), cellular components such as peroxisome and extracellular space (Fig. 5D), and molecular functions such as lipid binding and oleic acid binding (Fig. 5E).

### Validation of PPAR signaling pathway in rats

**Figure 6:**
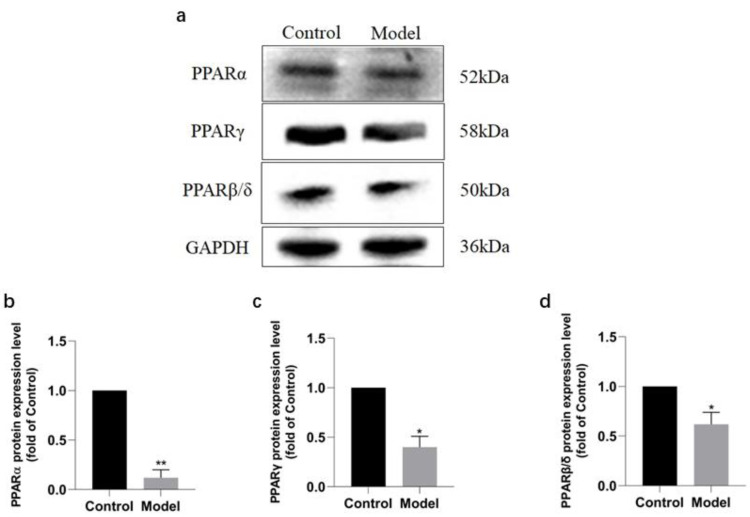
Expression levels of PPAR *α*, PPAR *γ*, and PPAR *β*/ *δ* between model and control group. (A) Western blots results of PPAR *α*, PPAR *β*, and PPAR *β*/ *δ* . (B) Statistical results PPAR *α* protein expression levels. (C) Statistical results PPAR *γ* protein expression levels. (D). Statistical results PPAR *β*/ *δ* protein expression levels.

Western blotting analysis revealed that the expressions of PPAR signaling pathway in the rats of model group were downregulated (Fig. 6A), with PPAR *α* most significantly decreased (*P* < 0.01) (91.2%, Fig. 6B), PPAR *γ* obviously decreased (*P* < 0.05) (63.60%, Fig. 6C), and PPAR *β*/ *δ* decreased (*P* < 0.05) (45.33%, Fig. 6D). The results were consistent with those of KEGG enrichment and GO annotation analysis of DEPs in the rats of two groups.

## Discussion

### Main findings

A rat model of HFpEF was established to make a comparative study with the control group, which has been confirmed to be a successful model in terms of behavioral changes, cardiac functions, and pathological changes of the rats. iTRAQ technology was used to identify 563 DEPs of the rats in two groups, including 243 up-regulated proteins and 320 down-regulated proteins. Among them 13 DEPs demonstrated the closest connection with PPAR *α*, PPAR *β*/ *δ* and PPAR *γ* based on KEGG enrichment analysis and they were mainly related to such biological process as fatty acid beta-oxidation, such cellular components as peroxisome, and such molecular functions as lipid binding based on GO annotation analysis. Western blotting analysis confirmed that the expressions of PPAR *α*, PPAR *β*/ *δ* and PPAR *γ* in model group were consistent with the results of bioinformatics analysis in our study.

### Interpretation

**Figure 7:**
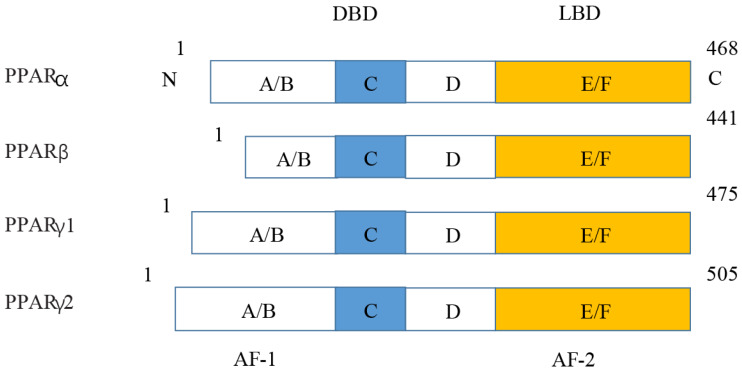
Molecular structure of PPAR *α*, PPAR *β*, PPAR *γ* 1, and PPAR *γ* 2.

PPARs were first discovered in 1990 and three members of the PPAR subfamily (PPAR- *α*, PPAR- *β*/ *δ* and PPAR- *γ*) have been found until now (Han et al., 2017b). PPARs include N/C terminal ligand-binding domain and DNA binding domain, which are divided into A/B region, C region, D region and E/F region according to their functions. A/B region is transcriptional activation region, C region is DNA binding region, D region is variable hinge region, and E/F region is ligand binding region (Han et al., 2017a) (Fig. 7). When PPARs are activated, they control the expression of many genes involved in energy metabolism and inflammation and regulate many metabolic pathways implicated in the pathogenesis of cardiovascular diseases (Han et al., 2017b). With the increasing incidence of HF in recent years, the study of metabolic regulation in the treatment of HF has been widely concerned (Heggermont et al., 2016; Zhang et al., 2019). As a major transcriptional regulator of energy metabolism, PPAR *α* is mainly expressed in the liver, kidney, and heart and it is essential for regulating inflammation and angiogenesis.

Our study has found that the LVEF index of the rats in model group was in line with the reported criteria for the HFpEF animal model (van Ham et al., 2022). Plenty of studies have investigated the characteristics of the same rat model in terms of physiological parameters, such as LVEF (Cho et al., 2018; Liu et al., 2021; Zhang et al., 2020) and they have provided powerful evidence for the efficient construction of HFpEF rat model in the present study. Furthermore, previous animal experiments have shown that the pathogenesis of HFpEF is the abnormal endothelial function in microcirculation induced by continuous low-grade inflammation and oxidative stress (Fukuta et al., 2021; Martin et al., 2018; Pitt et al., 2014) so PPARs could maintain the energy supply mode by up-regulating the expression of lipid metabolism (Fukuta et al., 2021). PPAR *α* could identify specific DNA sequences and regulate the expression of target genes by forming isodimers with retinoid X receptors. On the one hand, activation of PPAR *α* up-regulated the key enzymes in FA oxidation and promoted energy supply by enhancing *β* oxidation of cardiomyocytes (Kaimoto et al., 2017). On the other hand, activation of PPAR *α* in left ventricular cardiomyocytes reduced left ventricular dilation by promoting FA oxidation (Chuppa et al., 2018). In this manuscript, PPAR *α* was down-regulated when the rats suffered from HFpEF. When PPAR *α* acted on the downstream target genes, it prevented myocardial apoptosis by obstructing mitochondrial FA metabolism and reducing myocardial oxygen consumption. Since substrate utilization and intermediate metabolic disorders, energy deficiency and oxidative stress are the basis of HFpEF progression, ATP and PCR expressions significantly decreased. Previous studies have found that the overexpression of PPAR *α* in cardiomyocytes resulted in cardiac systolic dysfunction since it accelerated the glycogen deposition and apoptosis of cardiomyocytes and decreased their antioxidant capacity (Duerr et al., 2014). It was observed that oxidative dysfunction of cardiac FA and HFpEF occurred in PPAR *α*-deficient mice (Lam et al., 2015) so PPAR *α* agonists could delay the progression of HFpEF by promoting FA oxidation to provide the necessary energy for myocardium (Elezaby et al., 2015).

PPAR *β*/ *δ* plays a pivotal role in regulating FA metabolism. Previous experiments have shown that upregulating PPAR *β*/ *δ* promoted mitochondrial generation and inhibited metabolic remodeling in stress-induced HF, thereby protecting cardiac function (Liu et al., 2011). In recent years, many enzymes and proteins involved in lipid and energy metabolism have been identified as direct targets of PPAR- *β*/ *δ* (Mansour, 2014). The synthesis of such PPAR- *β*/ *δ* agonists as GW0742, GW501516, L165041, and GW1929 has been confirmed to be effective in the treatment of HFpEF (Bojic & Huff, 2013; Ding, Yang & Yang, 2014; Ehrenborg & Krook, 2009; Mansour, 2014; Plutzky, 2011; Wagner & Wagner, 2010). Our study has found that activating PPAR *β*/ *δ* has affected lipid and energy metabolism and it is obvious that it has a protective effect on HFpEF.

Similar to PPAR *α* and PPAR *β*/ *δ*, PPAR- *γ* is involved in the regulation of cardiac metabolic function. Our study has found that activation of PPAR *γ* had a regulatory role in HFpEF though PPAR *γ* was only expressed at low and moderate levels. Previous studies have shown that activating PPAR *γ* inhibited the nuclear translocation of nuclear factor- *κ*B (NF- *κ*B) P65, reduced the expression of stress-related molecules in downstream pathways, decreased the oxidative stress level, and alleviated cardiac hypertrophy (Han et al., 2017b). The expression of PPAR *γ* was upregulated in ventricular biopsy specimens of patients and hypertrophic cardiomyopathy mice under certain pathophysiological conditions (Krishnan et al., 2009). Mir-130a played a positive role in angiotensin II fibrosis by regulating the expression of PPAR *γ*.

With the aging of global population, the proportion of HFpEF in HF is increasing year by year so the development of PPAR agonists with strong activity and high safety has become the focus of HFpEF research. Although the safety of beta, a small molecule of PPAR *α* agonist, has been reported in a meta-analysis, no studies have been conducted in the reduction of HF mortality by such beta drugs (Jakob et al., 2016). Thiazolidinediones are PPAR *γ* agonists. An increase in cardiac events associated with rosiglitazone administration has been observed in the past, which may be related to lipid toxicity caused by the accumulation of myocardial ceramides and FAs. This is the reason why the cardiac effects of thiazolidinediones are still of great controversy. Although PPAR *β*/ *δ* could promote FA metabolism, its therapeutic effects remain elusive. Currently, PPAR *α* and PPAR *γ* agonists have not been applied to the treatment of HF and PPAR *β*/ *δ* agonists have not been approved for clinical use. Our future research will explore whether PPAR agonists could be used as safe and effective targets for the treatment of HFpEF.

### Strength and limitations

Strengths of this study include the comprehensive proteomic analysis of the pathological mechanism of HFpEF and PPARs as the physiological master switches in the heart. It is the first time we investigated the pathological mechanism of HFpEF using proteomic analysis, which has some advantages over RNA sequencing and some other methods. Our study has confirmed that PPARs could regulate cardiac energy metabolism and are closely related to the occurrence and development of HFpEF. Therefore, PPARs may be a potential therapeutic target for HFpEF. Novel PPAR agonists could provide new opportunities in the intervention of cardiovascular diseases in clinical practice.

We acknowledge the limitations of this study. Although the rat model of HFpEF is a generally accepted model and has been used as an experimental model of heart failure (Klotz et al., 2006), it is a question whether the results of the experiments on rats could be translated into the patients suffering from HFpEF. Moreover, only male rats were used in the model of HFpEF. Although this is consistent with other studies using only male animals and it is feasible to make comparisons (Reue & Wiese, 2022), it would provide more evidence to support our findings if both male and female rats had been used in the study.

### Conclusion

In conclusion, this work has successfully established a rat model of HFpEF using high salt diet, which has provided a good animal model for the investigation into the pathogenesis of HFpEF. iTRAQ technique has been employed to identify the DEPs of HFpEF in the model group and control group for the first time. In addition, bioinformatics analysis has been made to reveal that HFpEF is related to the PPAR signaling pathway, which has been verified by Western blotting. Our finding that there is a link between the downregulation of PPAR family proteins with HFpEF may provide a theoretical basis for the effective treatment of HFpEF in clinical practice.

##  Supplemental Information

10.7717/peerj.15280/supp-1 Supplemental Information 1 ARRIVE guidelines 2.0

## Additional Information and Declarations

### Data Availability

The following information was supplied regarding data availability: The data is available at Zenodo: Hang Xu. (2023). Pathological mechanism of heart failure with preserved ejection fraction in rats based on iTRAQ technology. https://doi.org/10.5281/zenodo.7742900.
