# Supplementary material for: Pathological mechanism of heart failure with preserved ejection fraction in rats based on iTRAQ technology
Source: PeerJ. 2023 May 3;11:e15280. doi: 10.7717/peerj.15280 (PMC10163871; doi:10.7717/peerj.15280)
Supplement: Supplemental Information 1 [file peerj-11-15280-s001.docx]

NOTE: Please save this file locally before filling in the table, DO NOT work on the file within your internet browser as changes will not be saved. Adobe Acrobat Reader (available free [here](https://acrobat.adobe.com/uk/en/acrobat/pdf-reader.html)) is recommended for completion.


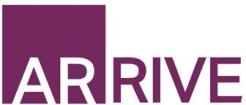
The ARRIVE guidelines 2.0: author checklist

| The ARRIVE Essential 10 | | | |
| --- | --- | --- | --- |
| These items are the basic minimum to include in a manuscript. Without this information, readers and reviewers cannot assess the reliability of the findings. | | | |
| **Item** |  | **Recommendation** | **Section/line number, or reason for not reporting** |
| **Study design** | 1 | Animals were placed on a 12:12 h light/dark cycle and received a regular granular diet. The rats were fed in SPF lab from Fourth Military Medical University and were given free access to food and water. They were placed in an animal chamber at room temperature 25°C±1°C, air humidity 60%. Rats were randomly assigned to each experimental group. Ten adult male salt-sensitive rats (180-200g) were purchased from Beijing Vital River Experimental Animal Science and Technology Co., Ltd. (SCXK 2016-0006). The rats were fed in the Experimental Animal Center of Air Force Medical University. They were placed in an animal chamber with room temperature 25°C ± 2°C, air humidity 50%-70%, and light/dark cycle for 12 h, and were given free access to food and water. After 7 days of adaptive feeding, the rats were randomly divided into control group and model group (n = 5). Then the rats in control group were fed with 0.3% of NaCl diet and the rats in model group were fed with 8% of NaCl high-salt diet for 8 weeks, respectively. | Part 2 ，Section 1 |
| **Sample size** | 2 | Ten adult male salt-sensitive rats (180-200g) were purchased from Beijing Vital River Experimental Animal Science and Technology Co., Ltd. (SCXK 2016-0006). The rats were fed in the Experimental Animal Center of Air Force Medical University. They were placed in an animal chamber with room temperature 25°C ± 2°C, air humidity 50%-70%, and light/dark cycle for 12 h, and were given free access to food and water. After 7 days of adaptive feeding, the rats were randomly divided into control group and model group (n = 5). Then the rats in control group were fed with 0.3% of NaCl diet and the rats in model group were fed with 8% of NaCl high-salt diet for 8 weeks, respectively. | Part 2，Section 1 |
| **Inclusion and exclusion criteria** | 3 | 1. Criteria including:LVEF≥50%、BNP＞100 pg/ml、NT-ProBNP＞300 pg/ml 2. No exclusions:Every rat met the criteria 3. For each analysis,n=5 | Part 2，Section 1 |
| **Randomisation** | 4 | 1. The rat number were accorded to measurements and animal/cage location. | Part 2，Section 1 |
| **Blinding** | 5 | Every author was aware of the situation at the different stages of the experiment. | Authors’ contributions |
| **Outcome measures** | 6 | 1. Subject to changes in values or observed results 2. Including body weight, running and swimming exhaustion time, cardiac functions, BNP and NT-proBNP, HE and Masson staining, Western blotting . | Part 2，Section 2、3、4、5、8 |
| **Statistical methods** | 7 | The experimental data were analyzed by SPSS 22.0 (Chicago, USA) and were presented as the mean ± standard deviation (SD). Comparison between two groups was made by two-tailed Student’s t-test. Statistical signiﬁcance was considered at p<0.05. | Part 2，Section 9 |
| **Experimental animals** | 8 | Ten adult male salt-sensitive rats (180-200g) were purchased from Beijing Vital River Experimental Animal Science and Technology Co., Ltd. (SCXK 2016-0006). | Part2，Section2 |
| **Experimental procedures** | 9 | 2.2 Measurement of body weight, running and swimming exhaustion time  The body weight of the rats was measured by electronic scale for the first time and then the rats were weighed every Monday for 8 weeks to observe the weight changes of the rats. The running and swimming exhaustion experiments were conducted to observe the physical changes of the rats after 8 weeks of feeding. The rats in both groups were placed on the treadmill and ran at 20 m/min until they reached the exhaustion standard (i.e., righting reflex disappeared). On the 2nd day of the running experiment, rats were placed in a water tank (water temperature 28±3℃, water depth 50 cm, length and width 50×50×80cm) and swam until they reached the exhaustion standard (i.e., their heads were submerged in water for 5 s and could not rise to the surface).  2.3 Detection of cardiac functions  After 8 weeks of feeding, the differences in cardiac functions of the rats in two groups were detected by echocardiography (X±SD, n=5). The rats were anesthetized through isoflurane inhalation. They were fixed in supine position on a table at 37°C with their hair on the left chest shaved. EF, left ventricular end systolic diameter (LVESD) and left ventricular end diastolic diameter (LVEDD) of the rats were detected by an ultrasound imaging system (RMV707B probe with a frequency of 30MHz and a detection depth of 12-16mm) with a 15L8 ultrasound probe. The probe was placed on the left chest of the rats and the left ventricular motion was recorded by M-mode ultrasound.  2.4 ELISA detection  After the blood of the rats was taken from abdominal aorta by vacuum vessels, it was put on ice and centrifuged within 30 m. Serum was collected and stored at -80°C for use. Then brain natriuretic peptide (BNP) was detected by ELISA kit (sensitivity: 0.75 pg/ml; Sigma Aldrich, USA) and N terminal pro B type natriuretic peptide (NT-proBNP) was detected by ELISA kit (sensitivity: 0.81 pg/ml; Abcam, USA). Each sample was repeated three times with the average value as the result. The OD value of absorbance was set as the ordinate (Y) and the corresponding standard concentration was set as the abscissa (X) to draw the standard curve. The sample content was calculated in line with the OD value (expressed as pg/ml) and the sample concentration was analyzed based on the standard curve.  2.5 HE staining and Masson staining  Heart tissues of the rats were administered with HE staining and Masson staining according to previous work[16]. Simply, heart tissues were cut with pathological methods and were observed with a microscope.  2.6 iTRAQ analysis  iTRAQ technology was applied to screen and identify DEPs of the rats in control group and model group. Analyses were made according to the established procedures in our laboratory[17] (Figure 1).  2.7 KEGG enrichment analysis and GO annotation of DEPs  KEGG enrichment analysis and GO annotation of DEPs were conducted using BGI analysis system (https://biosys.bgi.com/#/report/login). KEGG enrichment analysis was made for the relation of DEPs with signaling pathways by a two-tailed Fisher’s exact test. GO annotation analysis was made to classify the DEPs in PPAR signaling pathway into biological process, cellular component and molecular function.  2.8 Western blotting  The expression levels of PPARα, PPAR β/δ and PPARγ in heart tissues of the rats were analyzed by Western blotting according to previous method[18]. After sealed, the membrane was combined with PPARα (15540-1-AP, Wuhan Sanying Biotechnology Co., Ltd), PPAR β/δ (GB113450, Wuhan Sewell Biotechnology Co., Ltd.) and PPARγ (16643-1-AP, Wuhan Sanying Biotechnology Co., Ltd.) as primary antibody, respectively and it was incubated at 4℃ overnight. Then the membrane coupled with the secondary antibody (HRP, A21020, Wuhan Abbkine Biotechnology Co., Ltd.) was incubated at room temperature for 1.5 h. Enhanced ChemoLuminescence (ECL) was used to detect protein band, which was visualized on autoradiography films. ImageJ was applied to read the band grey value. | Part2，Section2-8 |
| **Results** | 10 | 3.1 Changes in body weight, running/swimming exhaustion time of the rats  The body weight of the rats in model group was decreased (294 ± 10.04 g) compared with that in control group (321.8±22.42 g) on the last day of the eighth week (Figure 2a, Table 1). The running exhaustion time of the rats in model group was shortened (224±35.07s) compared with that in control group (367±35.28s) (Figure 2b) while the swimming exhaustion time of the rats in model group was greatly shortened (148.8±19.38 s) compared with that in control group (317.8±36.17 s) (Figure 2c).  3.2 Changes in cardiac functions of the rats  Echocardiography detection revealed that LVPWs and LVEF values in model groupwere greatlydecreased than those in control group while IVSd, LVIDd, LVIDs, and LVPWd values in model group were higher than those in control group with only IVSs no obvious value change, which indicated concentric remodeling of LV (Figure 2d, Table 2).  3.3 Changes in biochemical indexes of the rats  ELISA detection showed a remarkable difference in the contents of BNP and NT-proBNP in the rats of two groups. Compared with those in control group (57.3855±4.0792, 115.5455±9.9917), BNP and NT-proBNP of the rats in model group (110.7114±5.1450, 368.8182±32.0747) were significantly increased (Table 3).  3.4 Pathological changes in heart tissues of the rats  Figure 2f shows HE staining results of heart tissues of the rats in two groups. The myocardial cells in control group were orderly arranged with basically the same nuclear size, no swelling and atrophy, with good continuity and relatively parallel distribution. The vascular wall structure was complete, thin and thick, and the vascular intima was regularly arranged, with normal vascular ring shape. In contrast, there were disordered myocardial cells with inconsistent nuclear size, tangled and thickened vascular wall structure, broken and dissolved myocardial fibers, severe vascular lesions, and thickened vascular basement membrane in model group.  Figure 2g shows Masson staining observation of heart tissues of the rats in two groups. Myocardial cells in control group were neatly arranged and there were a few blue collagen fibers in the interstitium of cells and around blood vessels. However, a lot of collagen fibers were distributed in the myocardial interstitium and many interwoven collagen fibers were observed around blood vessels in model group. The accumulation of collagen fibers was greatly increased and the degree of fibrosis was significantly aggravated.  3.5 Identification of DEPs in rats  563 DEPs of the rats in two groups were analyzed according to Log2\|Fold Change\|>1.2 standard (P<0.05) through LC-MS/MS analysis (Figure 3a). Among them 243 proteins including Podn, LOC498155, Sh3kbp1, Anxa7, Gfpt2 and Bsg were significantly up-regulated while 320 proteins such as Eng, Clu, App, Bpgm5, LOC103689996 and Mrps18b were down-regulated.  3.6 KEGG enrichment analysis and GO annotation of DEPs in PPAR signaling pathway in rats  KEGG enrichment analysis showed that DEPs were most closely associated with PPAR signaling pathway, including its three subtypes: PPARα, PPAR β/δ and PPARγ. In addition, they were related to such signaling pathways as complement and coagulation cascades, biosynthesis of unsaturated fatty acids, primary bile acid biosynthesis, fatty acid degradation, ascorbate and aldarate metabolism, arachidonic acid metabolism, retinol metabolism, insect hormone biosynthesis, and fatty acid metabolism (Figure 3b). GO annotation analysis showed that these DEPs enriched in PPAR signaling pathway were mainly related to biological processes such as fatty acid (FA) beta-oxidation and fatty acid metabolic process (Figure 3c), cellular components such as peroxisome and extracellular space (Figure 3d), and molecular functions such as lipid binding and oleic acid binding (Figure 3e).  3.7 Validation of PPAR signaling pathway in rats  Western blotting analysis revealed that the expressions of PPAR signaling pathway in the rats of model group were downregulated, with PPARα ranked at the top, followed by PPARγ, and PPAR β/δ at the bottom (Figure 4). The results were consistent with the results of KEGG enrichment and GO annotation analysis of DEPs in the rats of two groups. | Part3，Section1-7 |

| The Recommended Set | | | |
| --- | --- | --- | --- |
| These items complement the Essential 10 and add important context to the study. Reporting the items in both sets represents best practice. | | | |
| **Item** |  | **Recommendation** | **Section/line number, or reason for not reporting** |
| **Abstract** | 11 | Objective: Heart failure with preserved ejection fraction (HFpEF) is an emerging public health problem worldwide. Treatments for the patients with HFpEF are not satisfactory because there is no unified understanding of the pathological mechanism of HFpEF. This study aims at investigating the potential pathological mechanism for the effective diagnosis and treatment of HFpEF.  Methods: Ten adult male salt-sensitive rats (180-200 g) were divided into control and model groups. The rats in model group were fed with a high-salt diet (8% NaCl) to induce HFpEF for this comparative study. Behavioral changes, biochemical indexes, and histopathological changes of the rats were detected. Then iTRAQ technology combined with bioinformatics analysis was employed to study the differentially expressed proteins (DEPs) and their enrichment in signaling pathways.  Results: 563 DEPs were identified in the rats of HFpEF and controls, with 243 up-regulated and 320 down-regulated. Compared with those in control group, the expression of PPAR signaling pathway in the rats of model group was down-regulated, with PPARα ranked at the top, followed by PPARγ, and PPAR β/δ at the bottom. The DEPs enriched in PPAR signaling pathway were mainly related to such biological processes as fatty acid beta-oxidation, such cellular components as peroxisome, and such molecular functions as lipid binding.  Conclusions: NaCl high-salt diet is one of the factors to increase the incidence of HFpEF in rats. PPARα, PPARγ and PPAR β/δ might be the targets of HFpEF. The findings may provide a theoretical basis for the treatment of HFpEF in clinical practice. | Abstract |
| **Background** | 12 | Heart failure (HF) is a very common cardiovascular syndrome in the world. HF is classified into four types according to left ventricular ejection fraction (LVEF) in universal definition and classification of heart failure in 2021. They are HF with reduced EF (HFrEF), HF with mildly reduced EF (HFmrEF), HF with preserved EF (HFpEF), and HF with improved EF (HFimpEF). Among them HFpEF accounts for more than 40% of HF patients with high mortality and morbidity. Majority of HFpEF patients are elderly women accompanied by such complications as hypertension, atrial fibrillation and renal insufficiency They suffer from impaired left ventricular diastolic function, increased stiffness and pulmonary congestion, leading to dyspnea, pulmonary hypertension and exercise intolerance. Since HFpEF has different clinical phenotypes and complex pathophysiological mechanism, HFpEF patients are currently considered as the largest “unmet needs” in cardiology. Therefore, in-depth study of HFpEF pathological mechanism is essential for the effective treatment of HFpEF.  Previous research has explored the pathogenesis of HFpEF based on epidemiology, pathophysiology, and cytology. Studies have found that hypertension is a main cause of HFpEF and HFpEF is mainly correlated with left ventricular diastolic dysfunction, exogenous constraints and left ventricular filling pressure, left ventricular systolic dysfunction, left atrial dysfunction, pulmonary hypertension, right ventricular dysfunction, coronary microvascular dysfunction, vascular and microvascular dysfunction, and abnormal peripheral and cardiac amyloidosis. Some studies of the transgenic mice expressing catalase in mitochondria have showed that mitochondrial abnormalities, left ventricular hypertrophy and diastolic dysfunction induced by high fat and high sugar diet could be improved, indicating that mitochondrial reactive oxygen species may cause mitochondrial dysfunction to a certain extent. In high-fat and high-sugar MHD mice, reduced ATP production results in functionally important energy deficiency and increased ADP while maintaining CK flux. Nowadays there is no unified understanding of the pathogenesis of HFpEF and there are limitations in the understanding of HFpEF by clinicians. With the development of proteomics, it is necessary to take full play of the new science and investigate the pathological mechanism of HFpEF from a new perspective.  Proteomic technique is a reliable method to make quantitative analysis of proteins based on peptide identification. iTRAQ technology has been widely used in the study of many cardiovascular diseases in recent years but there are few reports of its application in HFpEF. We established a rat model of HFpEF by feeding SD rats with high-salt diet to make a comparison with healthy rats. iTRAQ technology combined with KEGG enrichment analysis and GO annotation was employed to study the pathogenesis of HFpEF. Our study aimed at providing a potential pathological mechanism for the effective diagnosis and treatment of HFpEF. | Part1 |
| **Objectives** | 13 | Heart failure with preserved ejection fraction (HFpEF) is an emerging public health problem worldwide. Treatments for the patients with HFpEF are not satisfactory because there is no unified understanding of the pathological mechanism of HFpEF. This study aims at investigating the potential pathological mechanism for the effective diagnosis and treatment of HFpEF. | Abstract |
| **Ethical statement** | 14 | The animal experiment was performed with the formal approval of the Medical Ethics Committee of the First Affiliated Hospital of the Fourth Military Medical University (number:KY20213001) and was in line with the Guidelines for the Management and Use of Laboratory Animals by the Chinese National Institutes of Health.  All the procedures in this study were in accordance with the Helsinki Declaration of 1975, as revised in 2000, and were approved by the ethical review board of Frist affiliated hospital of Fourth Military Medical University (FE15611-07). Consent to participate is NA. | Part2，Section1  Ethics approval and consent to participation |
| **Housing and husbandry** | 15 | The rats were fed in the Experimental Animal Center of Air Force Medical University. They were placed in an animal chamber with room temperature 25°C ± 2°C, air humidity 50%-70%, and light/dark cycle for 12 h, and were given free access to food and water. | Part2，Section1 |
| **Animal care and monitoring** | 16 | All rats which participated in the control and model groups were mercifully killed (excess CO_2_) whereas unparticipated mice were fed for other experiments. | Part2，Section1 |
| **Interpretation/ scientific implications** | 17 | 3.1 Changes in body weight, running/swimming exhaustion time of the rats  The body weight of the rats in model group was decreased (294 ± 10.04 g) compared with that in control group (321.8±22.42 g) on the last day of the eighth week (Figure 2a, Table 1). The running exhaustion time of the rats in model group was shortened (224±35.07s) compared with that in control group (367±35.28s) (Figure 2b) while the swimming exhaustion time of the rats in model group was greatly shortened (148.8±19.38 s) compared with that in control group (317.8±36.17 s) (Figure 2c).  3.2 Changes in cardiac functions of the rats  Echocardiography detection revealed that LVPWs and LVEF values in model groupwere greatlydecreased than those in control group while IVSd, LVIDd, LVIDs, and LVPWd values in model group were higher than those in control group with only IVSs no obvious value change, which indicated concentric remodeling of LV (Figure 2d, Table 2).  3.3 Changes in biochemical indexes of the rats  ELISA detection showed a remarkable difference in the contents of BNP and NT-proBNP in the rats of two groups. Compared with those in control group (57.3855±4.0792, 115.5455±9.9917), BNP and NT-proBNP of the rats in model group (110.7114±5.1450, 368.8182±32.0747) were significantly increased (Table 3).  3.4 Pathological changes in heart tissues of the rats  Figure 2f shows HE staining results of heart tissues of the rats in two groups. The myocardial cells in control group were orderly arranged with basically the same nuclear size, no swelling and atrophy, with good continuity and relatively parallel distribution. The vascular wall structure was complete, thin and thick, and the vascular intima was regularly arranged, with normal vascular ring shape. In contrast, there were disordered myocardial cells with inconsistent nuclear size, tangled and thickened vascular wall structure, broken and dissolved myocardial fibers, severe vascular lesions, and thickened vascular basement membrane in model group.  Figure 2g shows Masson staining observation of heart tissues of the rats in two groups. Myocardial cells in control group were neatly arranged and there were a few blue collagen fibers in the interstitium of cells and around blood vessels. However, a lot of collagen fibers were distributed in the myocardial interstitium and many interwoven collagen fibers were observed around blood vessels in model group. The accumulation of collagen fibers was greatly increased and the degree of fibrosis was significantly aggravated.  3.5 Identification of DEPs in rats  563 DEPs of the rats in two groups were analyzed according to Log2\|Fold Change\|>1.2 standard (P<0.05) through LC-MS/MS analysis (Figure 3a). Among them 243 proteins including Podn, LOC498155, Sh3kbp1, Anxa7, Gfpt2 and Bsg were significantly up-regulated while 320 proteins such as Eng, Clu, App, Bpgm5, LOC103689996 and Mrps18b were down-regulated.  3.6 KEGG enrichment analysis and GO annotation of DEPs in PPAR signaling pathway in rats  KEGG enrichment analysis showed that DEPs were most closely associated with PPAR signaling pathway, including its three subtypes: PPARα, PPAR β/δ and PPARγ. In addition, they were related to such signaling pathways as complement and coagulation cascades, biosynthesis of unsaturated fatty acids, primary bile acid biosynthesis, fatty acid degradation, ascorbate and aldarate metabolism, arachidonic acid metabolism, retinol metabolism, insect hormone biosynthesis, and fatty acid metabolism (Figure 3b). GO annotation analysis showed that these DEPs enriched in PPAR signaling pathway were mainly related to biological processes such as fatty acid (FA) beta-oxidation and fatty acid metabolic process (Figure 3c), cellular components such as peroxisome and extracellular space (Figure 3d), and molecular functions such as lipid binding and oleic acid binding (Figure 3e).  3.7 Validation of PPAR signaling pathway in rats  Western blotting analysis revealed that the expressions of PPAR signaling pathway in the rats of model group were downregulated, with PPARα ranked at the top, followed by PPARγ, and PPAR β/δ at the bottom (Figure 4). The results were consistent with the results of KEGG enrichment and GO annotation analysis of DEPs in the rats of two groups.  Limitations: Although the rat model of HFpEF is a generally accepted model and has been used as an experimental model of heart failure, it is a question whether the results of the experiments on rats could be translated into the patients suffering from HFpEF. Moreover, only male rats were used in the model of HFpEF, it would provide more evidence to support our findings if both male and female rats had been used in the study. | Part3，Section1-7  Part4, Section3 |
| **Generalisability/ translation** | 18 | Although the rat model of HFpEF is a generally accepted model and has been used as an experimental model of heart failure, it is a question whether the results of the experiments on rats could be translated into the patients suffering from HFpEF. | Part4, Section3 |
| **Protocol registration** | 19 | Before this study, we prepared an experimental scheme to study the potential pathogenic targets and signaling pathways of HFpEF, which were mainly verified by proteomics and Western blot. | NA |
| **Data access** | 20 | This study has the research data, which is in the authors. | NA |
| **Declaration of interests** | 21 | Conflict of interest  Author Tian Li is an editor of PeerJ and a statistical editor of Ann Med and have reviewed the statistical methods.  Fundings  This work was supported by the National Natural Science Foundation of China (82274313), the State Administration of Traditional Chinese Medicine of the People’s Republic of China (GZY-KJS-2021-004), Shaanxi Administration of Traditional Chinese Medicine (2020-ZXY-001), Key R&D Program of Shaanxi Province (2023GHZD43).  Ethics approval and consent to participation  All the procedures in this study were in accordance with the Helsinki Declaration of 1975, as revised in 2000, and were approved by the ethical review board of Frist affiliated hospital of Fourth Military Medical University (FE15611-07). Consent to participate is NA.  Authors’ contributions  Conceptualization: Hang Xu, Yi Ding, Jing Ma;  Methodology: Hang Xu, Xiaoqian Zhang, Kai Gao, Tian Li, Yi Ding, Jing Ma;  Software, Hang Xu, Xiaoqian Zhang, Kai Gao, Tian Li;  Validation: Hang Xu, Xiaoqian Zhang, Kai Gao, Tian Li, Yi Ding, Jing Ma; formal analysis: Hang Xu, Xiaoqian Zhang, Kai Gao;  Investigation: Hang Xu, Xiaoqian Zhang, Kai Gao;  Data curation: Hang Xu, Xiaoqian Zhang, Kai Gao, Tian Li, Yi Ding, Jing Ma;  Writing — original draft preparation: Hang Xu, Kai Gao;  Writing —review and editing: Hang Xu, Tian Li, Yi Ding, Jing Ma;  Supervision: Tian Li, Yi Ding, Jing Ma;  Project administration: Hang Xu, Tian Li, Yi Ding, Jing Ma;  Funding acquisition: Yi Ding, Jing Ma. | Conflict of interest、Fundings、  Ethics approval and consent to participation、Authors’ contributions |


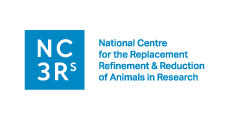
[www.ARRIVEguidelines.org](http://www.arriveguidelines.org/)
